# Hidden genetic diversity among *Blochmanniella* endosymbionts of closely related carpenter ant populations

**DOI:** 10.1093/jeb/voaf137

**Published:** 2025-11-18

**Authors:** Reo H Maynard, Yumary M Vasquez, Gordon M Bennett

**Affiliations:** Department of Life and Environmental Sciences, University of California, Merced, California, United States; Department of Energy Joint Genome Institute, Berkeley, California, United States; Department of Life and Environmental Sciences, University of California, Merced, California, United States

**Keywords:** carpenter ant, *Blochmanniella*, *Blochmannia*, endosymbiosis, genome evolution, molecular evolution, selection

## Abstract

Carpenter ants (Family Formicidae; Genus *Camponotus*) are a globally distributed, arboreal clade. They harbor an intracellular obligate bacterial endosymbiont known as “*Candidatus* Blochmanniella spp.” (hereafter *Blochmanniella*). The host ant species, *C. vicinus*, is geographically dispersed across the western United States of America and western Canada. To investigate how *Blochmanniella* have differentially evolved from related host-endosymbiont lineages, we sampled a *C. vicinus* population from California’s Sierra Nevada mountains, California, U.S.A., at an elevation of 2,300 m. Using morphological characters and Cytochrome Oxidase I markers, we determined that this population is genetically distinct from geographically distributed lineages of *C. vicinus* from Central California and Western North America (Arizona, U.S.A. to British Columbia, Canada). Thus, we sequenced the genome of the *Blochmanniella* endosymbiont from this host to understand how closely related symbiont lineages evolve. While our newly sequenced lineage is syntenic with other *Blochmanniella*, it has lost genes involved in membrane maintenance, bacterial cell information, and nutrition synthesis. Protein-coding genes across its genome are highly divergent as well (average sequence similarity = 93.6%). Therefore, we refer to our novel lineage as the *B. vicinus* Sequoia lineage (BSEQ). BSEQ can provide 7 of the 10 essential amino acids required by its insect host. It can also help break down toxic urea and repair UV radiation-induced DNA damage. Tests of selection reveal that most protein-coding genes BSEQ and related lineages are under strong or relaxed purifying selection. Taken together, our results demonstrate that while BSEQ and related *Blochmanniella* lineages have highly conserved content, there is considerable evolutionary diversity between them.

## Introduction

Carpenter ants (Formicidae: *Camponotus*) are a globally distributed genus of arboreal ants that preferentially inhabit fallen trees and manmade wooden structures (Rossi & Feldhaar, 2020). These species are generally omnivorous, preferring sugar-rich but nutrient-poor plant-based foods (Hölldobler & Wilson, 1990). To enhance dietary flexibility and support, carpenter ants have evolved an obligate endosymbiosis with the bacterium “*Candidatus* Blochmanniella spp.” (*Enterobacteriaceae*; hereafter referred to as “*Blochmanniella”*; Sauer et al., 2000). This provisional genus name was recently reclassified from “*Ca*. Blochmannia” to avoid nomenclatural confusion between the International Codes of Nomenclature for prokaryotes, algae, fungi, and plants (e.g., Oren et al., 2021). Nevertheless, *Blochmanniella* lives intracellularly within specialized cells punctuated throughout the ant midgut and ovaries for vertical transmission (Degnan et al., 2004; Kupper et al., 2016; Rafiqi et al., 2020; Sauer et al., 2002).

*Blochmanniella* provides a range of nutritional services to their ant hosts. Its primary role is believed to involve the provisioning of essential amino acids (EAAs; Gil et al., 2003; Williams & Wernegreen, 2015). Although *Blochmanniella* genomes are generally streamlined for EAA provisioning, they also encode other semi-essential and non-essential amino acids. For example, they widely retain the ability to synthesize tyrosine, which is required for proper insect exoskeleton formation and hardening hardening (Anbutsu et al., 2017). In addition to direct amino acid synthesis, *Blochmanniella* lineages retain the capability to recycle nitrogen through a cluster of urease genes (Konieczna et al., 2012; Williams & Wernegreen, 2010). This ability allows ant hosts to consume otherwise toxic animal wastes rich in urea and uric acid, which can help detoxify accumulated wastes and possibly supply the nitrogen necessary for amino acid synthesis (Feldhaar et al., 2007).

The carpenter ant-*Blochmanniella* symbiosis is ancient, having been established >40 million years ago (Manthey et al., 2022; Sauer et al., 2000; Wernegreen et al., 2009). Intracellular endosymbionts, like *Blochmanniella*, exist in small populations that are genetically isolated from the open environment with limited opportunities to acquire and exchange genes. They experience genetic bottlenecking with each host generation, as only a small subset of their populations is maternally inherited through transovarial processes (Bennett & Moran, 2015; McCutcheon et al., 2019; Perreau & Moran, 2022). Consequently, like other endosymbionts, *Blochmanniella* lineages have undergone dramatic gene losses with genomes ranging in size from 700 to 800 thousand base pairs (Degnan et al., 2005; Gil et al., 2003; Latorre & Manzano-Marín, 2017; Moran, 1996; Park et al., 2020). This gene loss stands in stark contrast to free-living relatives that typically range between four and five million base pairs (e.g., *E. coli* = 4.5 million bp, Blattner et al., 1997; *Salmonella enterica* = 4.9 million bp, McClelland et al., 2001; and *Pectobacteria cartovorum* = 4.8 million bp, Park et al., 2012). Given the effects of genetic drift in these systems, the genes that *Blochmanniella* lineages do retain continue to accumulate mutations that can affect their genetic identity, biochemical functionality, host interaction, and host-symbiont coevolution (Bennett & Moran, 2015; Boscaro et al., 2017; Perreau & Moran, 2022). Thus, different ant host species and populations are likely to have their own unique lineages of *Blochmanniella* with distinct evolutionary trajectories.

To better understand how distinct lineages of *Blochmanniella* have evolved across related ant hosts, we report on the endosymbiont isolated from a carpenter ant species restricted to the alpine environment of California’s Sierra Nevada Mountains, CA, U.S.A. The identified ant host is related to *Camponotus vicinus*, a geographically dispersed complex of distinct populations that occurs from the south rim of the Grand Canyon in Arizona to Sonoma Valley in California (e.g., Manthey et al., 2022; Ward et al., 2024). To identify our sampled *C. vicinus* ant host, we further investigated mitochondrial Cytochrome Oxidase I (COXI) divergence. While barcode analysis shows 100% matches to previously identified *C. vicinus* species, our Sierra Nevada population is 10% divergent from other genomically sequenced *Camponotus-Blochmanniella* systems that include *C. vicinus* from Sonoma, Central CA (e.g., Ward et al., 2024). This divergence indicates a distinct and previously uninvestigated host-symbiont population. Our comparative molecular evolutionary analyses of endosymbiont genomes widely recapitulate this divergence with unique molecular and genetic identities. Genes and their functional categories exhibit differential levels of molecular divergence and modes of selection, providing insight into how *Blochmanniella* lineages are evolving into distinct lineages, which can likely be generalized to other symbioses (Manthey et al., 2022). Given the genomic and genetic divergence between the newly sequenced lineage and others, we refer to our *Blochmanniella* lineage as *Blochmanniella vicinus* Sierra Nevada Mountain Sequoia strain (BSEQ).

## Materials and methods

### Carpenter ant sample collection, identification, and DNA extraction

Approximately 200 members of one carpenter ant colony were collected in Sequoia National Park in early June 2019 (36°34″57″N 118°44″56″W) (Figure S1). We ice-anesthetized twenty individual ants and removed their midguts intact in 1x PBS. Their pooled midguts (e.g., Melotto et al., 2023) were homogenized using a mini-bead mill (Avantor VWR) for 5 min at 3,100 rpm. DNA extraction was then performed using a PureLink Microbiome DNA Purification Kit (Thermo Fisher Scientific) following the manufacturer’s protocol. The resultant DNA concentration was measured using Qubit v.3 (Thermo Fisher Scientific). Metagenomic DNA was sequenced on an Illumina MiSeq for 4 million 2 × 300 base-pair paired-end reads at the qB3 Functional Genomics Laboratory, U.C. Berkeley, USA (vcresearch.berkeley.edu/research-unit/functional-genomics-laboratory).

### BSEQ genome sequencing and annotation

Genome assembly and genome annotation were performed with the Galaxy Project (www.useGalaxy.org; Jalili et al., 2020). To quality control raw reads, we first used Trimmomatic v.0.38.1 to trim sequencing adapters and low-quality bases (Phred score < 28; Bolger et al., 2014). Final read quality was checked with FASTQC v.0.11.8 (Simons, 2015). FASTQ Interlacer v.1.2.0.1 + galaxy0 was then used to combine forward and reverse reads (Blankenberg et al., 2010; Mehta et al., 2021). A *de novo* genome assembly of *Blochmanniella* was created from merged Illumina reads using Shovill assembler v.1.1.0 + galaxy1, with assembly executed via SPAdes v.3.14.1 (Prjibelski et al., 2020). Our initial assembly produced two bacterial chromosome fragments identified by their G + C content and shared deep-read coverage. The identities of these scaffolds were further confirmed using NCBI-BLAST for predicted protein-coding genes identified as *Blochmanniella* homologs (Johnson et al., 2008). The two scaffolds were merged using Geneious Assembler v.2023.2.1 (www.geneious.com). To assess genome assembly completeness and quality, reads were mapped back to the circularized *Blochmanniella* genome using Bowtie2 v.7.2.2 with default parameters (Langmead & Salzberg, 2012). No coverage breaks were observed throughout the entire circle. Average read coverage exceeded 5,000x.

The circular *Blochmanniella* genome was annotated using Prokka v.1.14.6 + galaxy1 (Seemann, 2014). Pseudogene annotation was performed with MetaGeneMark (Zhu, 2010) and further verified with NCBI-BLAST and by aligning against previously completed *Blochmanniella* genomes via Mauve (Degnan et al., 2005; Gil et al., 2003; Manthey et al., 2022; Park et al., 2020; Williams & Wernegreen, 2010). Ribosomal RNAs were identified with RNAmmer v.1.2 (Lagesen et al., 2007). The final genome organization, annotation completeness, and gene order of our *Blochmanniella* were checked against all other published complete *Blochmanniella* genomes available on NCBI (Table 1). The complete genome was uploaded to GenBank (NCBI; Benson et al., 2018) under accession number CP144371.

**Table 1.** NCBI GenBank accession numbers and RefSeq IDs of *Blochmanniella* whole genomes used in this study.

Lineage	GenBank assembly	RefSeq assembly	Genome assembly
**BTUR**	GCA_000973505.1	GCF_000973505.1	ASM97350v1
**BNPA**	GCA_014857065.1	GCF_014857065.1	ASM1485706v1
**BOBL**	GCA_000973545.1	GCF_000973545.1	ASM97354v1
**BVAF**	GCA_000185985.2	GCF_000185985.2	ASM18598v2
**BFLO**	GCA_000043285.1	GCF_000043285.1	ASM4328v1.1
**BNIP**	GCA_009827135.1	GCF_009827135.1	ASM982713v1
**B006 (C-006)**	GCA_023585745.1	GCF_023585745.1	ASM2358574v1
**BSEQ (SNP)**	GCA_036549215.1	GCF_036549215.1	ASM3654921v1
**BVIC**	GCA_030020825.1	GCF_030020825.1	ASM3002082v1
**B029 (C-029)**	GCA_023585905.1	GCF_023585905.1	ASM2358590v1
**B024 (C-024)**	GCA_023586085.1	GCF_023586085.1	ASM2358608v1
**B005 (C-005)**	GCA_023586525.1	GCF_023586525.1	ASM2358652v1
**B028 (C-028)**	GCA_023585925.1	GCF_023585925.1	ASM2358592v1
**B039 (C-039)**	GCA_023585845.1	GCF_023585845.1	ASM2358584v1
**B003 (C-003)**	GCA_023585685.1	GCF_023585685.1	ASM2358568v1
**B046 (C-046)**	GCA_023585865.1	GCF_023585865.1	ASM2358586v1
**B018 (C-018)**	GCA_023586365.1	GCF_023586365.1	ASM2358636v1
**B010 (C-010)**	GCA_023585705.1	GCF_023585705.1	ASM2358570v1
**B016 (C-016)**	GCA_023585765.1	GCF_023585765.1	ASM2358576v1
**B049 (C-049)**	GCA_023585825.1	GCF_023585825.1	ASM2358582v1
**B050 (C-050)**	GCA_023585725.1	GCF_023585725.1	ASM2358572v1
**BCHR**	GCA_000331065.1	GCF_000331065.1	ASM33106v1
**BPEN**	GCA_000011745.1	GCF_000011745.1	ASM1174v1
**BPEN7**	GCA_023016305.1	GCF_023016305.1	ASM2301630v1
**B036 (C-036)**	GCA_023585885.1	GCF_023585885.1	ASM2358588v1
**B019 (C-019)**	GCA_023586245.1	GCF_023586245.1	ASM2358624v1
**BMOD**	GCA_023585785.1	GCF_023585785.1	ASM2358578v1

Finally, to identify genes and functions shared by major *Blochmanniella* lineages, we compared the gene content of representative lineages that have fully sequenced genomes: *B. floridanus* (BFLO), *B. vafer* (BVAF), *B. nipponensis* (BNIP), *B. modoc* (BMOD), and *B. pennsylvanicus* (BPEN) and BSEQ. Genes were identified and binned into clusters of orthologous groups (COG) categories, and metabolic pathway completeness was determined with Kyoto Encyclopedia of Genes and Genomes (KEGG) (www.genome.jp/kegg;Tatusov et al., 2001).

### Molecular divergence and phylogenetic placement of BSEQ

**Figure 1.**
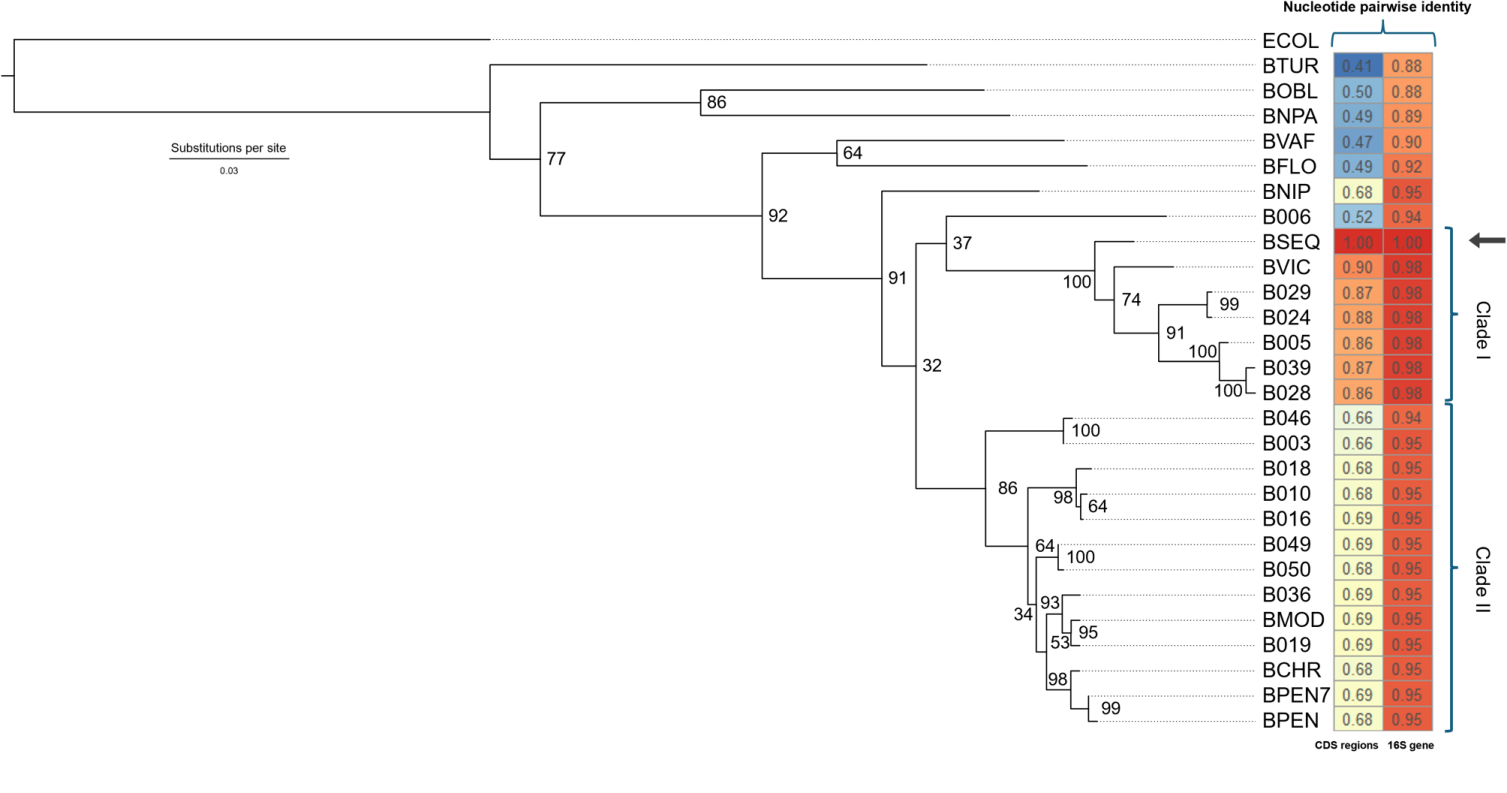
Phylogenetic tree of *Blochmanniella* lineages showing placement of a newly sequenced *B. vicinus* genome from California’s Sierra Nevada Mountains (BSEQ). Phylogeny was inferred using maximum likelihood approaches in RAxML for bacterial 16S rRNA gene. Bootstrap support for each relationship is shown at each node and estimated from 500 replicates. The heat map represents % divergence for 16S rRNA, and avg. % divergence for CDS relative to BSEQ. Black arrow shows the placement of the newly sequenced BSEQ lineage. Included lineages are those with publicly available genomes (see Table 1).

We obtained all complete *Blochmanniella* genomes available at GenBank, including the *Blochmanniella* from the genera *Polyrhachis* (i.e., BTUR, endosymbiont of *Polyrhachis*) and *Colobopsis* (i.e., BNPA, endosymbiont of *Colobopsis*; Table 1). All CDS orthologs for the sampled *Blochmanniella* genomes were then extracted and aligned in a global and pairwise (BSEQ vs. BVIC) fashion using the MAFFT L-INS-I model in version 7.525 (default settings; Katoh & Standley, 2013). Percent divergence for gene alignments was then calculated in Geneious 2025.0.3 (see Figures 1 and 3). We then reconstructed the phylogenetic relationships of *Blochmanniella* with 16S rRNA gene with *Escherichia coli* (ECOL) as an outgroup (*Gammaproteobacteria*: Gao et al., 2009; Williams et al., 2010). Alignments of the 16S rRNA gene were performed using Muscle v.5.1 in Geneious Prime v.2023.2.1 (Kearse et al., 2012). A phylogenetic tree was reconstructed using RAxML (Geneious plugin v.4.0) with rapid bootstrapping (500 replicates) and the “search for best-scoring ML tree” (“-f a -x 1”) parameters, employing the GTR GAA model for nucleic acid sequences (Stamatakis, 2014). The final tree was rendered and labeled using FigTree v.1.4.4.

### Determining patterns of selection between *Blochmanniella* lineages

To test for selection among *Blochmanniella* lineages, protein-coding genes were re-annotated using a common approach for all genomes with Prodigal v.2.6.3 (Table 1; default parameters; Hyatt et al., 2010). Single-copy orthologs were identified with OrthoFinder v.2.5.5 (Emms & Kelly, 2019). Similarity searches were then performed with DIAMOND v.2.1.9 in ultra-sensitive mode (Buchfink et al., 2015). Multiple sequence alignments were generated with MAFFT v.7.525 (Katoh & Standley, 2013). Orthogroups that contained exactly one gene from every genome were retained. For each retained orthogroup, coding sequences and MAFFT alignments were used with PAL2NAL v.14 (Suyama et al., 2006) to obtain codon-level alignments required for evolutionary analyses.

Tests of selection among lineages were undertaken with the branch model of CODEML in PAML v.4.10.9 (omega = 0.2, kappa = 2, NSsites = 0, model = 0 or 2; Yang, 2007). Likelihoods of a null model with a single ω for all branches of the tree and an alternative model that assigns separate ω values to the focal branches (e.g., the branches within the *B. vicinus* Clade I; Figure 1; Figure S2). The alternative model assigned separate ω values to BSEQ (branch #1) and BVIC (branch #2), while additional ω were assigned to the B029–B028 clade (#3), for B006 (#4), and for the branch leading to the clade (#5), the rest of the tree was considered background branches (see Figure S2 for complete labeling). To determine the best-fit branch model, we used a likelihood ratio test to compare the null model and the alternative model (2 * Δln*L*). Synonymous (dS) and nonsynonymous (dN) rates from orthologs that were significantly improved using the alternative model were extracted for plotting (see *Results* and *Discussion* below). Finally, genes in the tested orthogroups were functionally reannotated with eggNOG-mapper v.2.1.12 (Cantalapiedra et al., 2021), providing COG assignments for downstream interpretation (see Figure 3 legend for definitions of COG categories).

### Statistical analysis of divergence between COG

To determine if COG categories show elevated divergence between BSEQ and BVIC lineages, we conducted an ANOVA with post-hoc pairwise comparisons of our COGs using Tukey’s Honest Significant Difference (HSD) test. To account for false discovery rates (FDR), the HSD test (*p* < .05) was employed, which controls for family-wise error rates, which is a more conservative correction method than FDR tests (Bourget, 2025). All statistical analyses were performed using R (2024.12.0 Build 467). Statistical significance for each of our tests was carried out at a *p* < .05 significance level.

## Results and discussion

### Ant host and *Blochmanniella* endosymbiont identification

Our target ant species is morphologically similar to *C. vicinus*, which is described from California, Utah, and Arizona, U.S.A. (Mackay, 2019). We further used the Barcode of Life Data Systems (i.e., BOLD) to molecularly identify our sampled population (settings: Animal Species Level Database and Exhaustive Search; Ratnasingham & Hebert, 2007). The top 100 closest matches (% identity = 96%–100%) were *C. vicinus*. The highest matches (>99%) spanned populations from the Eastern California coastal mountains (e.g., Mt. Tamalpais) and British Columbia, Canada. Based on full-length mitochondrial Cytochrome Oxidase I species barcoding, this population is related to other California *C. vicinus* populations (e.g., Sonoma, CA, U.S.A.; Ward et al., 2024), but is approximately 10% divergent from those with available *Blochmanniella* genomes (see Figure 1; Figure S3). Furthermore, we observe high levels of molecular divergence between our newly sequenced *Blochmanniella* genome and others sequenced from *C. vicinus* host populations (see below). Taxonomic clarity is lacking for our considered population, as noted previously by Ward et al. (2024). Nevertheless, the genetic divergence observed in our study indicates that these populations are distinct from those of other previously investigated *Camponotus*-*Blochmanniella* systems (Hendrich et al., 2010; Ward et al., 2024). Therefore, we consider it to be a novel *B. vicinus* Sequoia strain from the Sierra Nevada Mountains (hereafter BSEQ), following naming conventions of adopting host species names for *Blochmanniella* lineages.

### *Blochmanniella* phylogenetic relationships

To determine BSEQ’s relationship with other *Blochmanniella* lineages, we reconstructed their phylogenetic relationships using bacterial 16S rRNA (Figure 1). We extracted the *Blochmanniella* 16S gene from 26 *Blochmanniella* lineages that have completely sequenced genomes available (NCBI; Table 1). The results show that BSEQ is closely related to the previously sequenced BVIC lineage (Bootstrap = 100; Manthey et al., 2022; Ward et al., 2024). However, BSEQ represents an earlier diverging lineage, sharing a common ancestor with the clade containing all other *C. vicinus* complex-associated endosymbionts. The average nucleotide identity for 16S rRNA between BSEQ from Sequoia National Park, CA, and BVIC from Sonoma, CA is 90%, with an average % divergence of 87.3% for all other *Blochmanniella* (Figure 1). Thus, BSEQ represents a divergent and distinct *Blochmanniella* lineage.

### Characteristics of the complete BSEQ genome reveal conserved cellular and nutritional roles among *Blochmanniella* lineages

**Figure 2.**
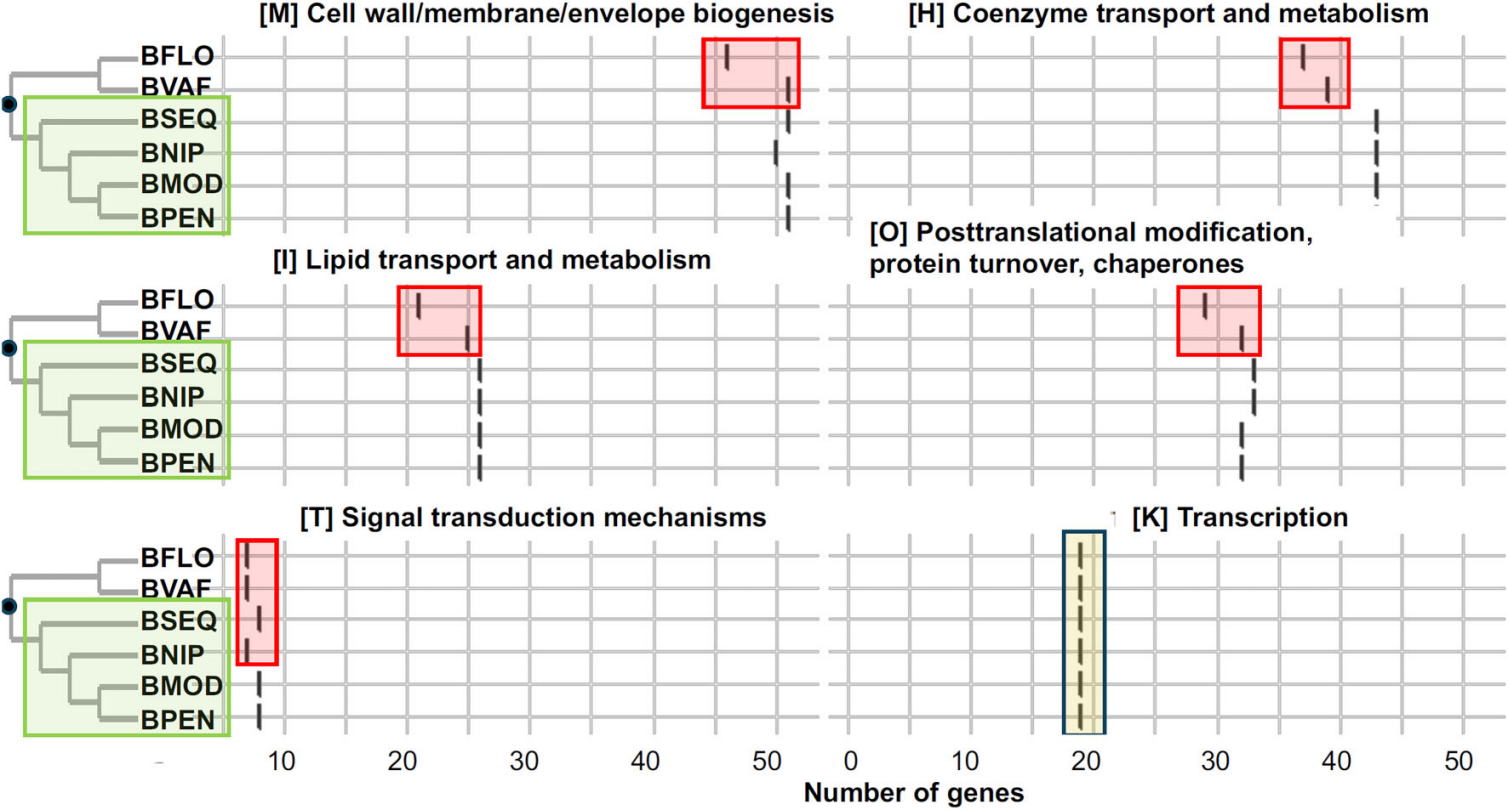
Differential gene losses in representatives from Clusters of Orthologous Genes (COG) categories for *Blochmanniella*. Graphs show gene count within COG categories for select divergent *Blochmanniella* lineages. Boxes show gene losses and retention. Relationship based on the 16S rRNA gene phylogeny (see Figure 1).

To characterize the evolutionary history and functional capacity of BSEQ, we sequenced its complete circular genome, which is 783,876 base pairs long and encodes 614 protein-coding genes (CDSs), one ribosomal cassette, and 39 transfer RNAs (tRNAs) that correspond to all 20 amino acids. The BSEQ genome is highly syntenic with other representative *Blochmanniella* lineages (i.e., BFLO, BVAF, BNIP, BMOD, BPEN; Figure 2; Supplemental Table 2). They share a core set of 561 genes (e.g., Degnan et al., 2005) that encode the capabilities for mRNA transcription (COG category K), posttranslational protein modification via chaperones and heat shock proteins (COG O), and protein translation with tRNA support for all 20 amino acids (COG J). They are further capable of performing central metabolisms, including the citric acid cycle (COG C) and a sulfate reduction capability (COG P) for amino acid synthesis (Gil et al., 2003), and urease recycling (see Figure 2; Figure S4). Compared with more closely related sister lineages, *Blochmanniella* BSEQ is 3,651 bp larger than BVIC, with approximately 54.6% of this difference (1,994 bp) residing within intergenic regions. The remaining 45.4% (1,657 bp) is located within open reading frames and accounts for most of the predicted gene length differences between the two lineages (Ward et al., 2024).

BSEQ likely provides similar metabolic services to those of other *Blochmanniella* lineages (e.g., Williams & Wernegreen, 2015). Its primary role appears to involve the provision of EAAs (Figure 2). Regarding EAAs, it encodes the pathways for histidine, isoleucine, leucine, lysine, methionine, threonine, and tryptophan (Figure 2). These are generally highly conserved across all available *Blochmanniella* lineages. However, some of these pathways are missing precursor genes (see *Results* and *Discussion* below). The synthesis and provisioning of EAAs is a primary function of most obligate endosymbionts of insects, which have played a significant role in their evolution and diversification (Bennett et al., 2024; Kikuchi, 2009).

Like all other available *Blochmanniella* genomes, BSEQ retains capabilities to synthesize six non-EAAs, including glycine, which is relatively unusual for ancient nutritional endosymbionts with smaller genomes (McCutcheon & Moran, 2012; Moran & Bennett, 2014). While the retention of non-EAAs may be due to a lack of evolutionary time leading to their loss as seen in more ancient symbionts (McCutcheon et al., 2019), their persisting retention suggests some functional importance or benefit to the system. For example, among the complete non-EAAs retained by BSEQ and other *Blochmanniella* is tyrosine. Some insects with hardened, thick exocuticles, like weevils, harbor symbionts that also retain the ability to synthesize tyrosine (Anbutsu et al., 2017). In these insects, tyrosine supplied by bacterial endosymbionts is essential and involved in strengthening and darkening exoskeletal structures (de Souza et al., 2011; Moriyama & Fukatsu, 2022; Santos-Garcia et al., 2017). Thus, we predict that *Blochmanniella* non-essential amino acid supplementation similarly plays a crucial role in the darkening of carpenter ant exoskeletons, which is vital for their survival in difficult and arid environments (Dong et al., 2024).

BSEQ and other *Blochmanniella* lineages further retain key capabilities essential for cellular and environmental persistence. First, BSEQ encodes the ability to import glucose and mannose as primary energy sources through a complete phosphoenolpyruvate-dependent phosphotransferase pathway (Zientz et al., 2004). Second, BSEQ’s genome retains the *uvrD* gene, which is essential for repairing UV radiation (UVR) damage (Williams & Wernegreen, 2015). Its retention may aid in nucleic acid protection and repair in its host’s alpine environment. BSEQ, for example, is found in carpenter ants inhabiting elevations greater than approximately 2,100 m and exposed to UVR levels that are elevated by 11%–19% compared to sea level (Blumthaler et al., 1997). While the functional analysis of *uvrD* and its potential as a UVR damage repair gene remains to be investigated, it suggests distinct ways in which the host’s ecology may shape endosymbiont genomes.

### Gene losses in BSEQ and related *Blochmanniella* lineages

Ongoing genome erosion has occurred across evolutionary scales, including between divergent *Blochmanniella* lineages and between closely related *B. vicinus* populations. Among more distantly related lineages, gene losses have occurred in a wide range of COG categories, although these losses differ between lineages and clades (Figure 2; Supplemental Table 2). At the broadest level, BSEQ and related lineages (BNIP, BPEN, and BMOD) retain more genes and complete COG categories than other lineages (Figure 2; Manthey et al., 2022; Williams & Wernegreen, 2015). In contrast, related BFLO and BVAF lineages have differentially lost genes involved in COG functions that include cell envelope biogenesis (COG M), Lipid transport and metabolism (COG), coenzyme transport and metabolism (COG H), and posttranslational modification (COG O), among other gene losses (Figure 2). These distinct gene losses in BFLO and BVAF continue a trend of losing cell envelope capabilities (COG M), which further include peptidoglycan cell wall maintenance (*htpX*), membrane upkeep (*engB*), and protein modification and export (*elyC, secB*). Gene losses in BFLO and BVAF have also impacted essential metabolisms in amino acid synthesis, including the loss of coenzyme A synthesis genes (in both) and glutamine synthesis (specific to BVAF; Koga & Moran, 2014; Parish et al., 2022). The observed gene losses in *Blochmanniella* may be expected as more ancient insect endosymbionts rarely retain them, and these metabolisms are instead provided by hosts (Kaltenpoth et al., 2025; McCutcheon & Moran, 2012).

At a finer evolutionary scale, *Blochmanniella* populations also show ongoing gene loss. The BSEQ lineage from the California Sierra mountains distinctly retains a more complete ability to independently synthesize ubiquinone, whereas BVIC from the California’s Sonoma region has lost several essential genes in the pathway (e.g., *ubiH ,visC*). In bacteria, ubiquinone plays essential roles in electron transport in *E. coli* and other bacteria (Kwon et al., 2000), functions that may no longer be essential in some *Blochmanniella*. Moreover, there have been some gene losses in EAA pathways in BSEQ and other *Blochmanniella*. For example, all *Blochmanniella* lineages are missing gene *lysC*, which in *E. coli* catalyzes the first step in the biosynthesis of isoleucine, lysine, and threonine (Figure 2). All lineages are also missing gene *ilvBN*, which catalyzes the first step in leucine production, as well as an intermediate step in isoleucine production (Figure 2). We interpret these universal losses to be ancient ones because they occur in all of the carpenter ant clades we surveyed. Species BOBL and BNPA are also missing *ilvA*, which catalyzes a different intermediate step in the isoleucine biosynthesis pathway (Figure 2).

The BSEQ genome further contains several small predicted open reading frames, likely resulting from relatively recent deletions of gene segments or insertion-deletion frameshift mutations (Figure S5). We interpret these ORF fragments as non-functional gene remnants. Several putatively inactivated genes include *hisH, lptG, rluD*, and *ybiS*. The loss of these genes likely influences interactions between the *C. vicinus* hosts and the Sierra Nevada BSEQ symbiont, particularly in relation to bacterial-host cell interactions via membrane structures, bacterial cell information processing, and nutritional synthesis and exchange. Briefly, the histidine synthesis factor *hisH* has become a pseudogene in BSEQ, BVIC, and several other *Blochmanniella* lineages (e.g., BCHR, BMOD; see Figure S5). Histidine synthesis can likely still proceed without *hisH* if provided with high levels of ammonia (Chioccioli et al., 2020; Rieder et al., 1994). Some ant-*Blochmanniella* symbioses may obtain abundant ammonia through a host diet rich in urea-based compounds from animal wastes. In contrast, the inactivation of *lptG* and *ybiS*—which are important for anchoring peptidoglycan and the transport of lipopolysaccharide, respectively—play important roles in cell-envelope integrity in BSEQ (Asmar et al., 2017; Narita & Tokuda, 2009; Sperandeo et al., 2008). Finally, the loss of *rluD* can result in cell growth defects due to the breakdown of rRNA and tRNA biosynthesis pathways, which are essential cellular processes in bacteria (Gutgsell et al., 2005). We assume that the loss of *rluD* has been rescued by host-supplied or other bacterial support genes, which now perform these functions (e.g., Del Campo et al., 2004; Shigenobu et al., 2000).

### Genome-wide molecular divergence between *Blochmanniella* lineages

Our results reveal two distinct *Blochmanniella* clades, designated *B. vicinus* Clade I and *B. pennsylvanicus* Clade II (Figure 1), named after prominent species described within them. The *B. vicinus* Clade I lineages are ∼98% similar to BSEQ at the 16S gene locus and about 87.3% identical to BSEQ within orthologous CDS regions. The more-divergent *B. pennsylvanicus* Clade II lineages are about 95% similar to BSEQ at the 16S gene locus and about 68% similar to BSEQ within orthologous CDS regions (Figure 1). These findings highlight the generally conserved nature of the 16S rRNA gene relative to the more mutable CDSs.

**Figure 3.**
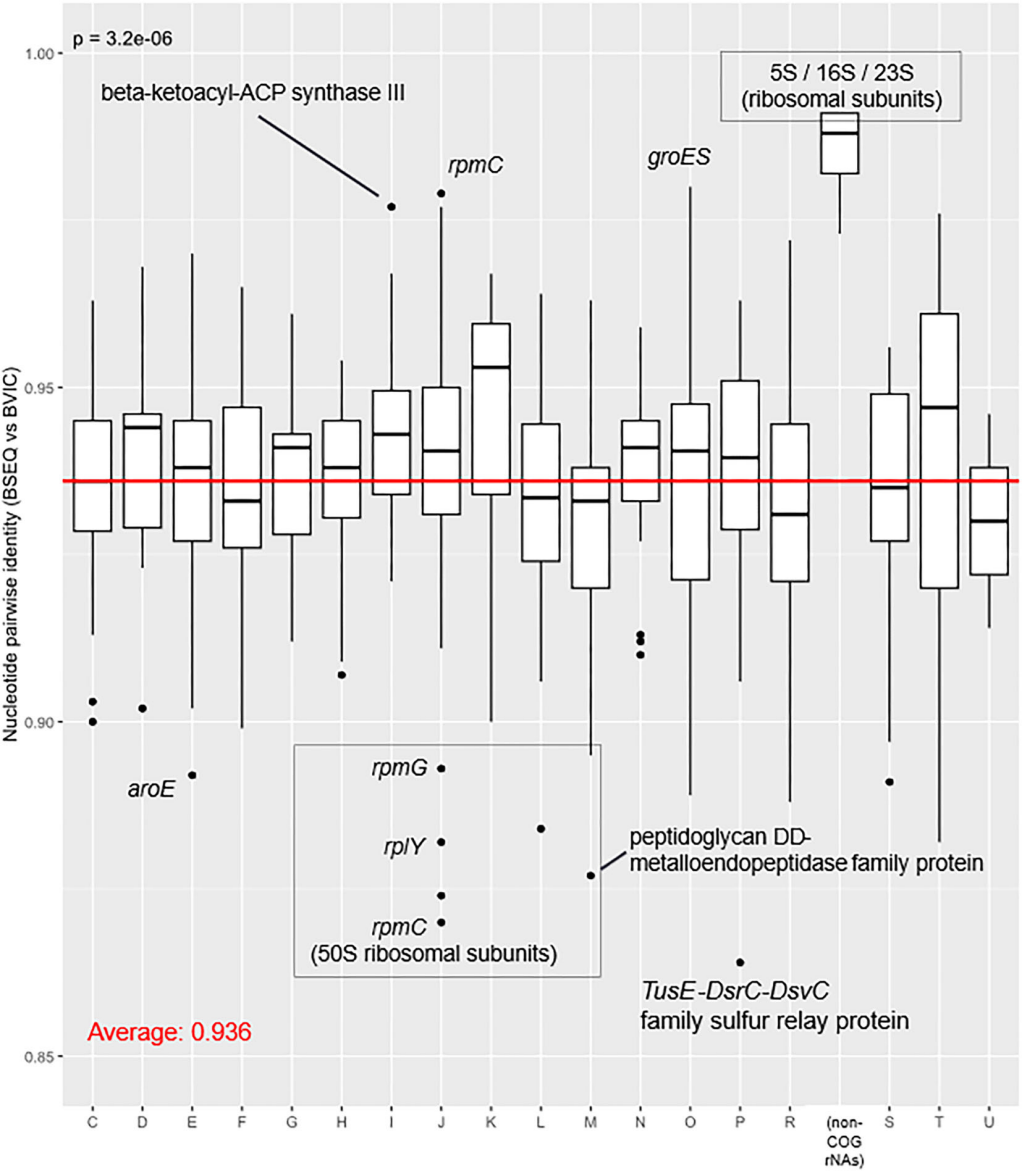
Comparison of changes in nucleotide identity between *B. vicinus* lineages from Sierra Nevada Mountains (BSEQ) and Central California (BVIC). The graph is organized by clusters of orthologous genes (COG): C—Energy production and conversion; D—Cell cycle control, cell division, chromosome partitioning; E—Amino acid transport and metabolism; F—Nucleotide transport and metabolism; G—Carbohydrate transport and metabolism; H—Coenzyme transport and metabolism; I—Lipid transport and metabolism; J—Translation, ribosomal structure and biogenesis; K—Transcription; L—Replication, recombination and repair; M—Cell wall/membrane/envelope biogenesis; N—cell motility; O—Posttranslational modification, protein turnover, chaperones; P—Inorganic ion transport and metabolism; R—General function prediction only; S—Unknown function; T—Signal transduction mechanisms; U—Intracellular trafficking, secretion and vesicular transport. COG categories, A—RNA processing and modification and B—Chromatin structure and dynamic, are not shown due to near complete loss of genes in these categories. A value of “1.00” indicates 100% nucleotide sequence homology.

To gain a deeper insight into how genes evolve between lineages, we compared genome-wide molecular divergence between Sierra Nevada BSEQ and the previously sequenced Central California BVIC lineages (see Figure 1; Manthey et al., 2022; Figure 3). We analyzed base pair sequence identity (i.e., divergence) across coding sequences (CDSs) and rRNA genes (see Figure 1 and Supplemental Table 1). Our results show that, on average, CDSs and rRNA genes are 93.6% and 98.5% similar, respectively. Pairwise identities between individual genes range from 86.4% (sulfur transfer protein, *tusE*) to 98.0% (heat shock protein, *groES*). We then tested whether gene functional categories (i.e., COG) show significant differences in their divergence. Since genetic drift is expected to be a major driver of endosymbiont genome evolution, it is reasonable to predict that similar divergence across genes and COGs should be observed (Bennett & Moran, 2015; Herbeck et al., 2003; Sabater-Muñoz et al., 2017; Tatusov et al., 2001). Deviations from that pattern indicate that some mode of selection is acting on those genes (Huelsenbeck et al., 2006). Analysis of variance showed significant differences in nucleotide pairwise identity among COG categories (ANOVA: *p* = .0000032, *F* = 1.872; see Figure S6). However, our post-hoc Tukey’s HSD test was unable to distinguish statistically significant COG categories. Thus, while differences in divergence between genes occur, we are unable to distinguish them by COG category alone. We note that COG categories A—RNA processing and modification and B—Chromatin structure and dynamics were excluded from our analyses since *Blochmanniella* only retains one gene in each (*prmB* [COG A] and *hns* [COG B]).

Despite a lack of statistical difference for specific COG categories, genes do show wide differences in their % divergence. These differences may indicate distinct modes of molecular evolution that drive their retained variation and shape their evolutionary function (e.g., purifying and relaxed selection, gene conversion, incomplete lineage sorting, etc.). Not surprisingly, genes that generally show the highest sequence identity include core functional and housekeeping genes. For example, among the genes showing the most conserved rates of molecular evolution are several ribosomal subunit genes (discussed below) and core housekeeping genes. The 5S, 16S, and 23S rRNA genes have conserved % similarities between BSEQ and BVIC of 99.1%, 98.5%, and 97.3%, respectively. These genes are essential in symbiont protein synthesis, which is intrinsic to most cellular functions. Similarly, bacterial *groL* and *groES* genes also have high levels of sequence conservation (similarity = 95.7% and 98%, respectively). Both of these genes assist in the folding and function of other proteins under stress (Fares, 2015; Kupper et al., 2014). In endosymbionts like *Blochmanniella*, GroL and GroES are regarded as essential intracellular support mechanisms that literally hold together crucial proteins that have undergone extensive mutations through persistent genetic drift (Barman et al., 2023; Fan et al., 2013; Kupper et al., 2014).

In contrast, genes with comparatively low levels of sequence similarity may be subject to relaxed purifying selection or other evolutionary forces (see *Patterns of selection between Blochmanniella lineages* below). For example, *Blochmanniella* genes involved in intracellular trafficking, secretion, and vesicular transport (COG U) are among the most divergent (avg. similarity = 93%; Figure 3). These genes are rarely retained in the smaller, older genomes of endosymbionts from other insect hosts (reviewed by Moran & Bennett, 2014). Similarly, several ribosomal accessory proteins, including *rplY, rpmC*, and *rpmG*, exhibit distinctly higher divergence levels compared to other genes in their COG J category (avg. similarity = 88.2%, 87.0%, and 89.3%, respectively; Figure 3). It is common for other endosymbionts to lose certain ribosomal accessory proteins (Moran & Bennett, 2014). Ribosomal cofactors can function interchangeably with the 50S ribosomal subunit (Seffouh et al., 2024), which may help to explain divergent rates of genes involved in ribosomal structures.

### Patterns of selection between *Blochmanniella* lineages

To determine how different modes of selection shape genes among *Blochmanniella* lineages, we examined genome-wide synonymous to non-synonymous ratios (dN/dS) with a branch model approach focusing on BSEQ and North American *Blochmanniella* lineages from related *C. vicinus* hosts (described above). To categorize and interpret the varying modes of selection that can operate on endosymbiont genomes, we followed the binning criteria described by Perreau & Moran (2022; see also for application): dN/dS < 0.1 indicates strong purifying selection where deleterious amino-acid changing substitutions are rapidly removed; dN/dS > 0.1 indicates relaxed purifying selection where slightly deleterious amino-acid changing substitutions can accumulate due to drift weakened selection; dN/dS ≥ 1.0 indicates positive selection where amino-acid changing substitutions confer adaptive benefits and are retained (although drift can also drive elevated dN/dS rates; (Kjeldsen eta al., 2012).

**Figure 4.**
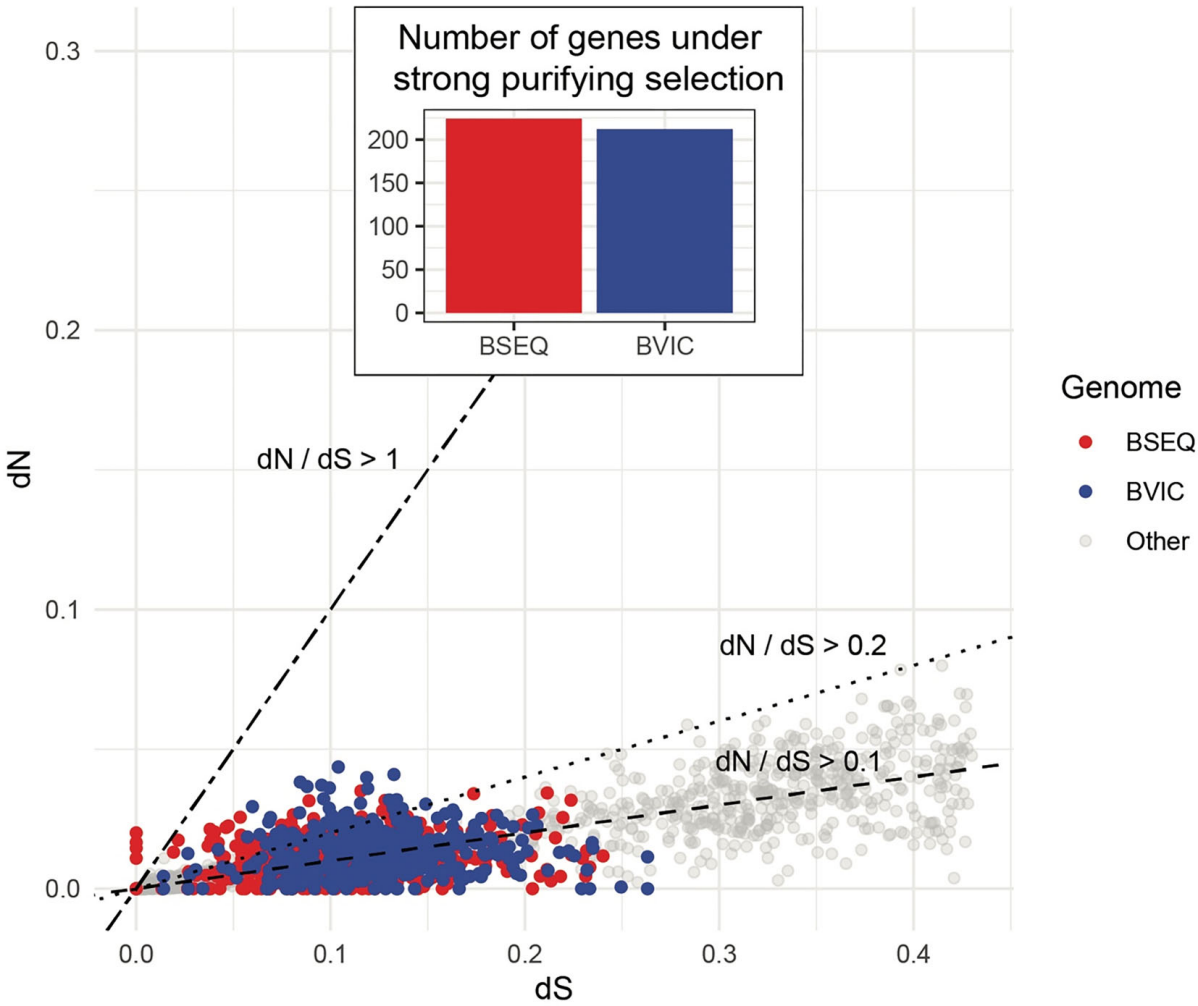
Genome-wide synonymous (dS) vs. non-synonymous (dN) substitution rates and selection among *Blochmanniella* lineages. The dN and dS rates were estimated for 471 single-copy orthologous genes shared across all available *Blochmanniella* genomes (see Table 1). Inset graph shows the total number of genes in BSEQ and BVIC under strong purifying selection. Rate estimates were inferred using CODEML under a branch model test of selection. Dashed lines show degrees of purifying and positive selection: dN/dS < 0.1 indicates strong purifying selection, dN/dS > 0.1 indicates relaxed purifying, and dN/dS ≥ 1.0 indicates positive selection (Perreau & Moran, 2022).

Across our surveyed *Blochmanniella* genomes, we identified 471 single-copy orthologous genes shared by every species. Likelihood ratio tests revealed lineage-specific patterns of selection for 424 genes, which fit the branch-specific model significantly better than a single-rate model (see Figure S2). Among the significant cases, 224 genes on the BSEQ branch and 212 on the BVIC branch displayed strong purifying selection (ω < 0.1; 152 genes shared between BSEQ and BVIC; Figure 4; Supplemental Table 2). When sites with dS = 0 (which resulted in an infinite ω) were removed, ω estimates spanned 0.00–0.81 for BSEQ and 0.00–0.49 for BVIC. Of all genes, 132 exhibited strong purifying selection in only one of the two lineages (60 in BVIC and 72 in BSEQ), demonstrating heterogeneity in strength of selection between BSEQ and BVIC (Figure 4).

To illustrate the potential functional impact of drift and relaxed selection, we provide a brief overview of some illustrative genes that exhibit elevated dN/dS ratios while not shpwing signatures of positive selection (dN/dS < 1) in BSEQ. Such genes may be interpreted as having been released to drift due to their reduced functional importance in the symbiosis (Perreau & Moran, 2022). Many of those genes on the higher end are also typically lost from more ancient endosymbionts (McCutcheon & Moran, 2012). For example, the reactive intermediate imine deaminase A gene *ridA* (dN/dS = 0.81) plays a critical role in degrading imines before they become reactive and harmful (Niehaus et al., 2015). Similarly, although the genes *rpmF* (dN/dS = 0.76), *trmD* (dN/dS = 0.69), and *rplU* (dN/dS = 0.56) encode key proteins and co-factors that support ribosome biogenesis and function (Aseev et al., 2016; Hori et al., 2017; Shirikawa et al., 2023), the loss of ribosomal subunits is not uncommon. Their functions may be no longer required; other ribosomal subunits can assume their roles, their functions are no longer needed in symbiont ribosomes, or hosts contribute their own genes to fill the gaps (Moran & Bennett, 2014). Finally, phosphocarrier protein HPr, encoded by *ptsH* (dN/dS = 0.50), is under moderate purifying selection. This gene is involved in the transport of sugars into bacterial cells, a function that is rarely retained in more ancient endosymbionts (McCutcheon et al., 2019).

In contrast to these genes that experience relaxed selection, some core functional and housekeeping genes in BSEQ are under strong purifying selection (Supplemental Table 2). For example, the protein elongation factor gene, *tufA*, exhibits one of the strongest signatures of purifying selection (dN/dS = 0.0001). The protein encoded by this gene plays a crucial role in tRNA binding to ribosomes, a fundamental function in protein synthesis (Kacar et al., 2017). The strong conservation of this gene’s molecular identity in BSEQ is likely due to its essentiality in maintaining the integrity of translational processes. Similarly, the gene encoding SsrA binding protein, *smpB*, which is a widely conserved bacterial protein involved in releasing stalled peptides from ribosomes, is under strong purifying selection (dN/dS = 0.0001; Karzai et al., 1999). Such a gene is likely critical to maintaining translational functions in the genomes of endosymbionts, including BSEQ, which are known to experience varying levels of drift and biased mutations (Moran, 1996). These molecular processes may compromise the function of complex holoenzymes and the ability of ribosomes to efficiently transcribe and translate highly mutated genes. Finally, the chaperonin gene *groL* in BSEQ also exhibits strong purifying selection (dN/dS = 0.0086). The GroL protein in endosymbionts is widely recognized for its critical role in supporting the folding, structural integrity, and function of other highly mutated proteins and enzymes (reviewed by Kupper et al., 2014). It is also among the most highly expressed genes in endosymbionts associated with insects across orders and families (Kupper et al., 2014). We assume this gene plays a similar role in BSEQ, given that it is under purifying selection and its wide functional retention in other insect symbioses.

## Conclusion

The western American carpenter ant, *C. vicinus*, inhabits the mountainous forests that extend across Arizona, California, Colorado, Nevada, Utah in the U.S. and into British Columbia, Canada (Manthey et al., 2022; Ward et al., 2024). The *C. vicinus* population and its *Blochmanniella* endosymbiont lineages analyzed here are genetically distinct. While we refer to our genetically distinct *Blochmanniella* as BSEQ, based on ecological, morphological, and molecular evidence as a member of the *C. vicinus* complex (Manthey et al., 2022; Ward et al., 2024), future host-level taxonomic work may be required to update host species boundaries. Nevertheless, although the BSEQ lineage is highly syntenic with other *C. vicinus*-associated *Blochmanniella* and other lineages more widely, genome-wide patterns of molecular evolution present a different story. *Blochmanniella* BSEQ from California’s Sierra Nevada mountains is highly divergent across most loci, with some essential symbiont genes exhibiting higher levels of sequence similarity (e.g., *groL* chaperonin, ribosomal accessory proteins, ATP synthase-associated genes). No genes appear to be under positive selection. They are instead either under strong purifying selection to maintain essential functions, or weak and relaxed purifying selection indicative of elevated drift (Moran, 1996). Thus, although there is strong structural and functional conservation of *Blochmanniella* genomes, our analyses reveal relatively high levels of hidden diversity among *Blochmanniella* endosymbionts and differening strengths of selection (Manthey et al., 2022). This cryptic diversity provides insight into how the carpenter ant*-Blochmanniella* endosymbiosis is actively evolving.

## Supplementary Material

voaf137_Supplemental_Files

## Data Availability

The newly sequenced genome for the “Candidatus *Blochmanniella* vicinus” Sequoia strain (BSEQ) has been deposited in NCBI’s GenBank genomic database (GenBank ID GCA_036549215.1) as “Blochmannia SNP” (Sequoia National Park).
