# Supplementary material for: Hidden genetic diversity among Blochmanniella endosymbionts of closely related carpenter ant populations
Source: J Evol Biol. 2025 Nov 18;39(1):158–69. doi: 10.1093/jeb/voaf137 (PMC12779916; doi:10.1093/jeb/voaf137)
Supplement: voaf137_Supplemental_Files [file voaf137_supplemental_files.zip › Supplemental Data.docx]

**SUPPLEMENTAL DATA**

|  |
| --- |
| 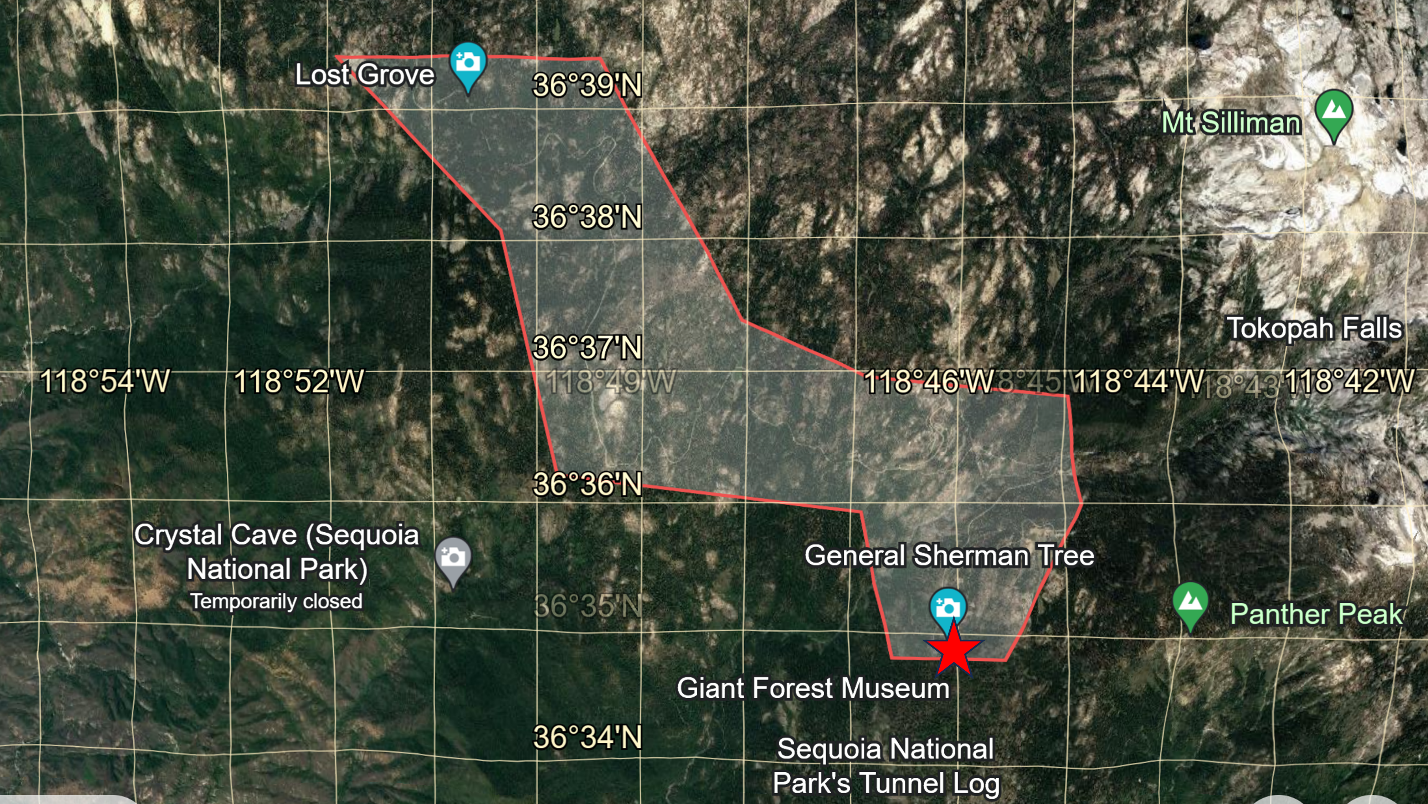 |
| **Figure S1. Camponotus *vicinus-*like carpenter ants in the Sierra Nevada Mountains (“*Camponotus sequoiaensis”*) hosting the BSEQ endosymbiont.** Discovery range (shaded area, main image above) straddles the boundary between Sequoia National Park and Sierra National Forest (boundary not indicated). It is likely that *C. sequoiaensis* carpenter ants are more widely distributed throughout California’s Southern Sierra Nevada Mountain Range than indicated here. Image credit: Google Earth. |

| **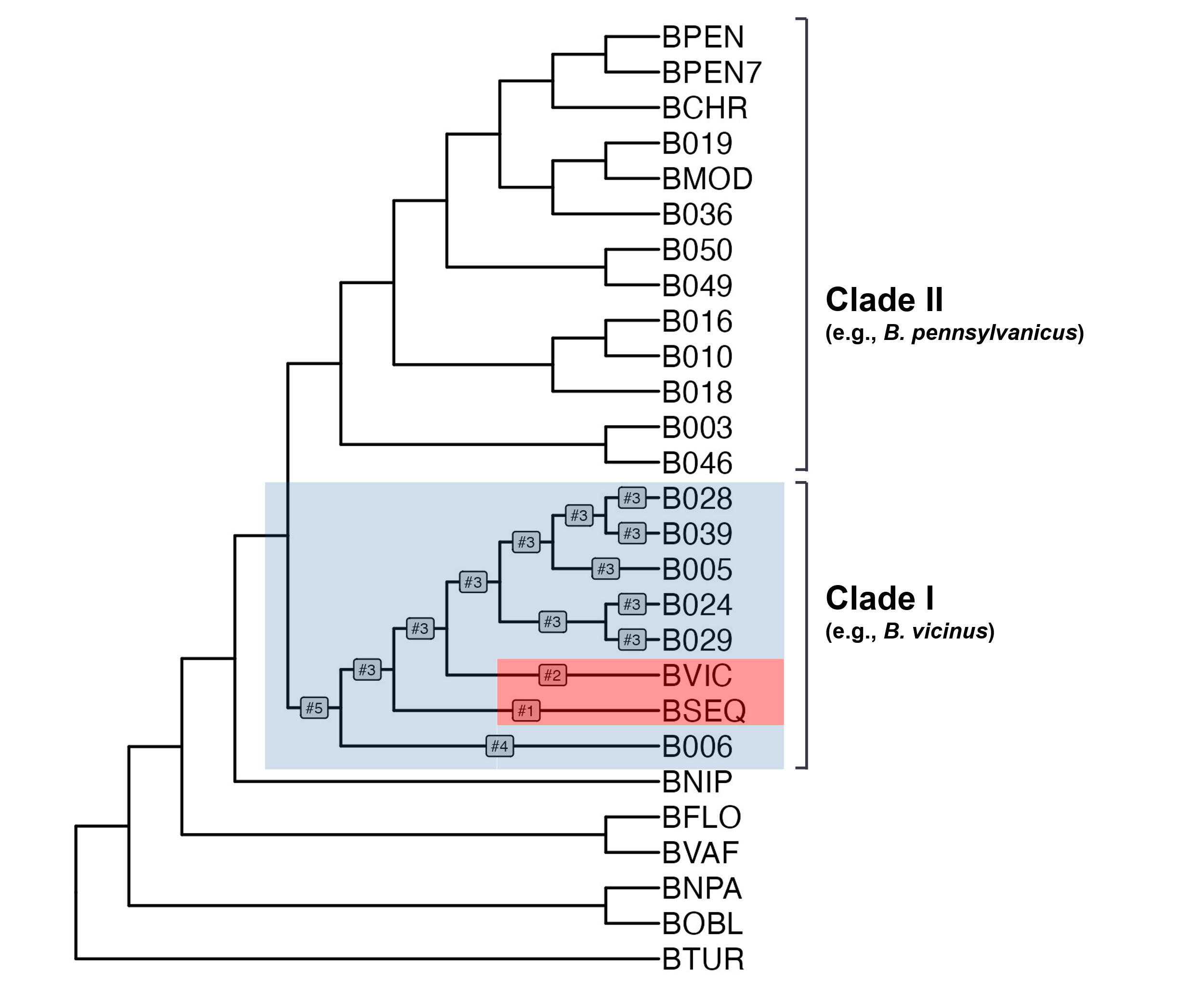** |
| --- |
| **Figure S2. Bayesian phylogeny for *Blochmanniella* of C. *vicinus*-like carpenter ants.** Clade I is highlighted in blue. Outgroup (ECOL) not shown. |

|  |
| --- |
| 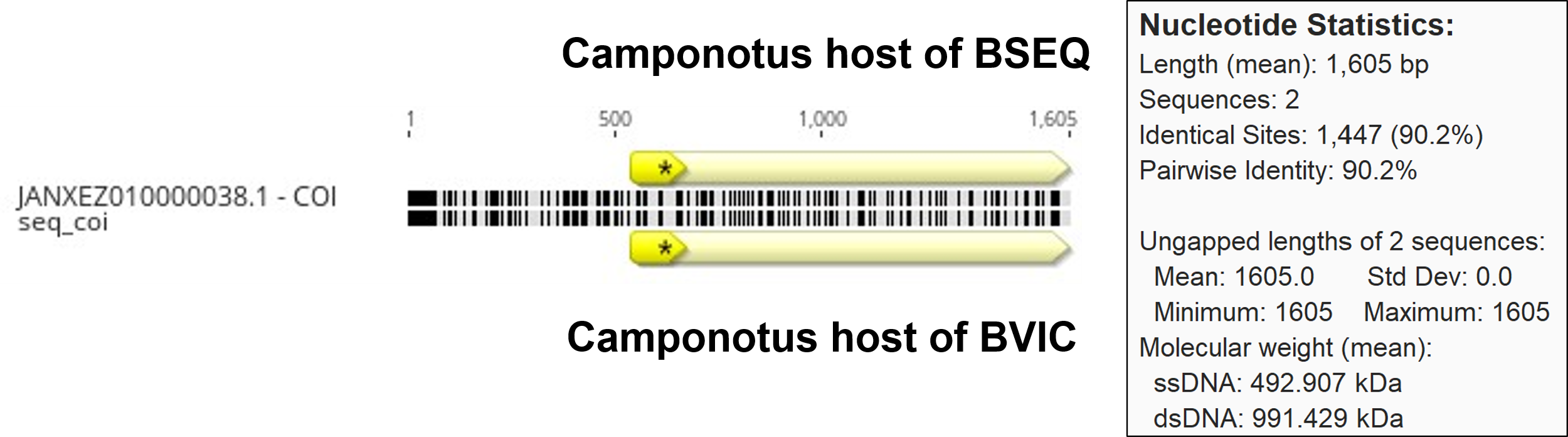 |
| **Figure S3. Cytochrome oxidase I dissimilarities between the BVIC host and BSEQ host.** Screenshot taken from Geneious Prime (Build 2024.11.22). |

| 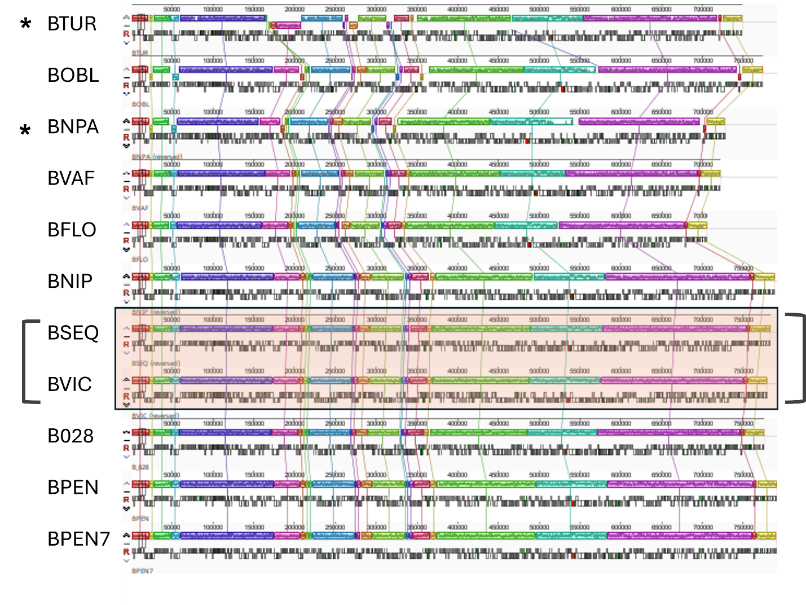 |
| --- |
| 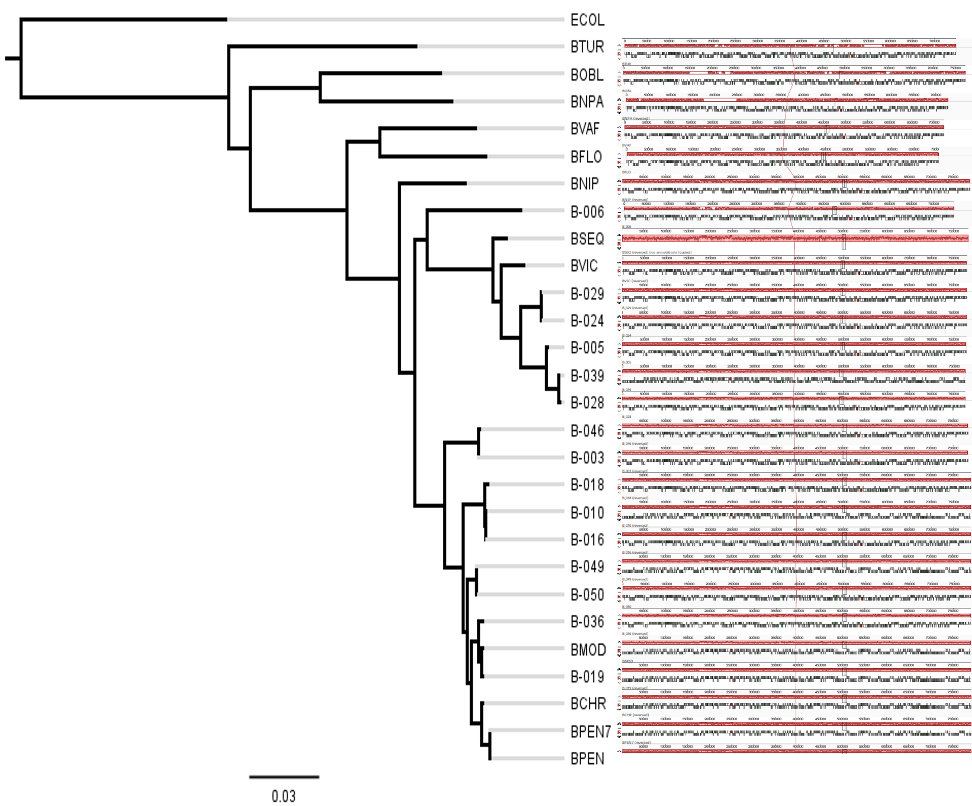 |
| **Figure S4. Genome synteny among certain *Blochmanniella* strains. Top panel. Worldwide *Blochmanniella* representation across Tribe Camponotini ants.** BTUR, Australia; BOBL, Eastern USA; BNPA, South Korea; BVAF, Southwestern USA; BFLO, Southeastern USA; BNIP, South Korea; BSEQ, Southwestern USA; BVIC, Southwestern USA; B028, Western USA; BPEN, Northeastern USA; BPEN7, Midwestern USA. Asterisk (*) indicates non-Camponotus host ants, but Tribe Camponotini nonetheless. **Bottom panel.** Unaligned syntenic map showing phylogenetic relationship between the strains used in this study. Image distortion (*Blochmanniella* strains) is for space economy. (Mauve; Darling et al., 2004). |

| 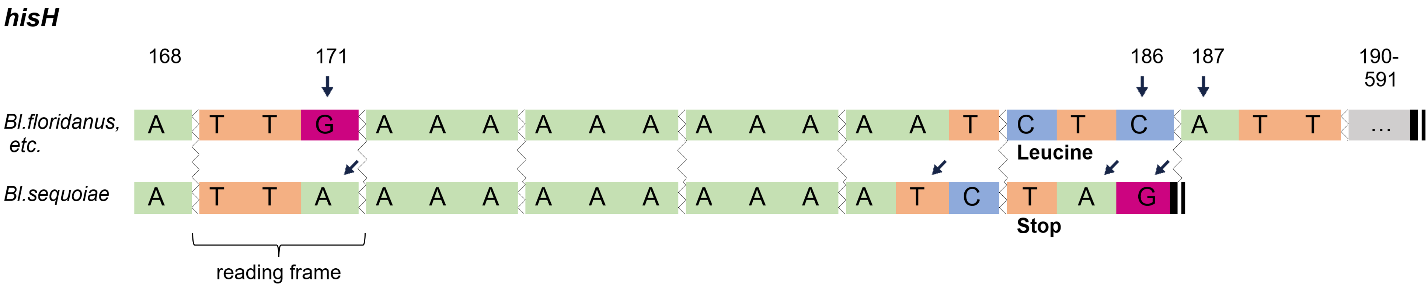 |
| --- |
| **Figure S5. Representative pseudogenization of a gene in BSEQ.** Truncation of histidine biosynthesis gene *hisH*: A frame shift (diagonal arrows) caused by a nucleotide deletion (G, position 171), coupled with two SNPs ( C.186>A.185 and A.187>G.186 ) led to a premature stop codon in a hypothetical BSEQ ancestor (here identified as *Bl. sequoiae*). |

| 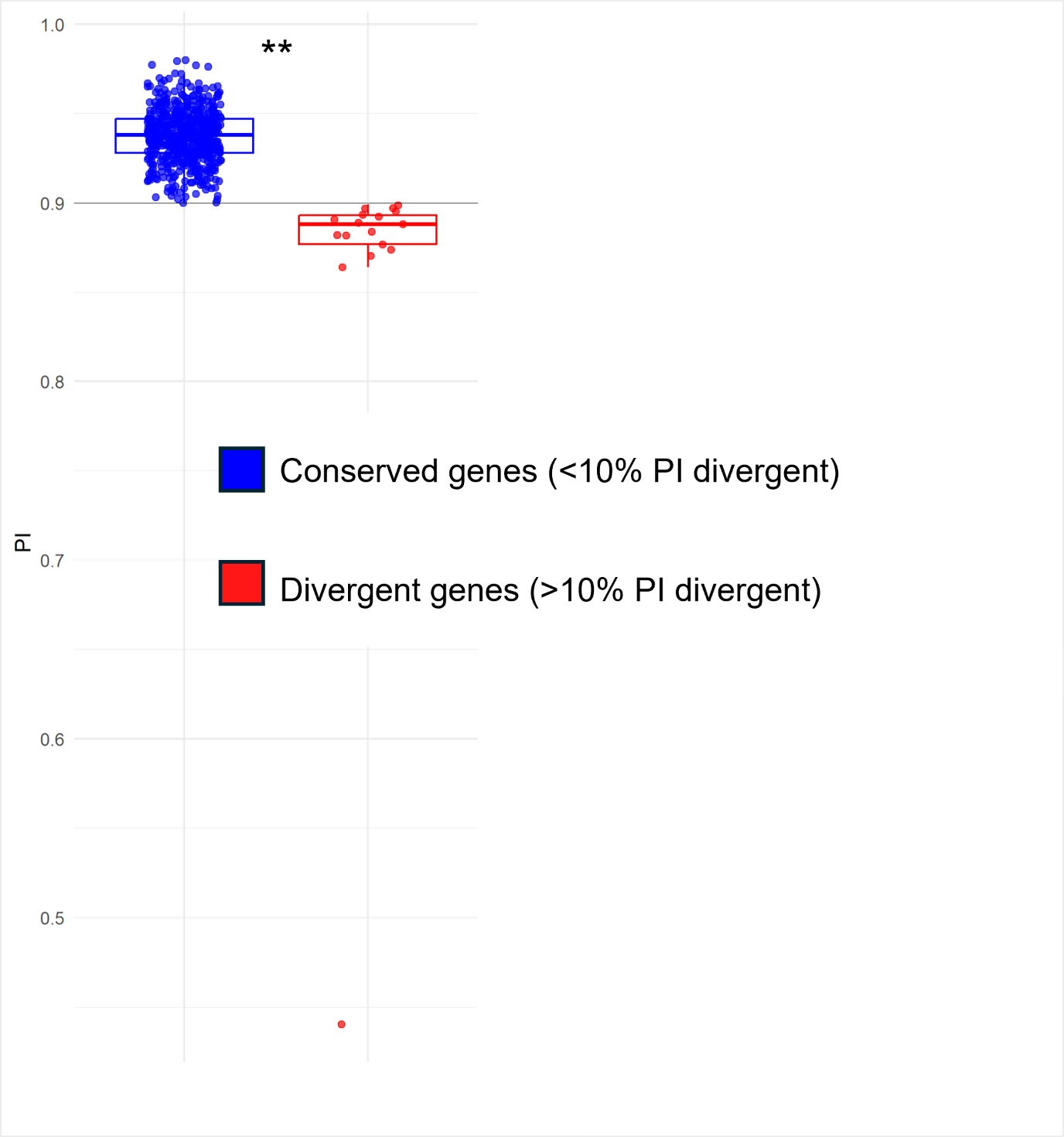 |
| --- |
| **Figure S6. Statistical difference between the divergent genes and the conserved genes in BSEQ and BVIC.** Statistical difference in the genes with greater than 10% difference in nucleotide pairwise identity. ANOVA: p = 0.0000032, F = 1.872. |
