# Supplementary material for: Hidden genetic diversity among Blochmanniella endosymbionts of closely related carpenter ant populations
Source: J Evol Biol. 2025 Nov 18;39(1):158–69. doi: 10.1093/jeb/voaf137 (PMC12779916; doi:10.1093/jeb/voaf137)
Supplement: voaf137_Supplemental_Files [file voaf137_supplemental_files.zip › Supplemental Table 1 - Pairwise Identity (BSEQ vs BVIC).docx]

| **Supplemental Table S1. Pairwise identity between homologous genes (BSEQ vs BVIC)** | | |
| --- | --- | --- |
| Gene Name | PI | COG |
| *prmB* | 0.944 | A |
| *ndhC* | 0.9 | C |
| *nuoN* | 0.903 | C |
| PhoH family protein | 0.913 | C |
| *cyoD* | 0.914 | C |
| *sbcB* | 0.916 | C |
| oxidative damage protection protein | 0.917 | C |
| *yfaE* | 0.919 | C |
| *sdhD* | 0.92 | C |
| 4-phosphoerythronate dehydrogenase | 0.922 | C |
| *atpF* | 0.925 | C |
| *odhB* | 0.927 | C |
| *nuoE* | 0.927 | C |
| F0F1 ATP synthase subunit epsilon | 0.929 | C |
| succinate dehydrogenase iron-sulfur subunit | 0.931 | C |
| *nuoK* | 0.931 | C |
| ferredoxin--NADP-plus reductase | 0.931 | C |
| *fldA* | 0.932 | C |
| 2-oxoglutarate dehydrogenase E1 component | 0.933 | C |
| *ppa* | 0.934 | C |
| *lpdA* | 0.934 | C |
| *fumC* | 0.934 | C |
| *atpB* | 0.934 | C |
| *nuoM* | 0.935 | C |
| NADH-quinone oxidoreductase subunit B family protein | 0.936 | C |
| *aceF* | 0.936 | C |
| *gpsA* | 0.937 | C |
| *sucC* | 0.939 | C |
| *nuoG* | 0.939 | C |
| *cyoC* | 0.939 | C |
| *cyoA* | 0.94 | C |
| *nuoH* | 0.941 | C |
| *nuoL* | 0.943 | C |
| *nuoI* | 0.943 | C |
| *aceE* | 0.944 | C |
| *sucD* | 0.945 | C |
| *nuoJ* | 0.945 | C |
| F0F1 ATP synthase subunit delta | 0.945 | C |
| *rsmI* | 0.947 | C |
| *sdhA* | 0.948 | C |
| *atpD* | 0.948 | C |
| *nuoF* | 0.95 | C |
| *atpE* | 0.95 | C |
| *atpA* | 0.95 | C |
| *nuoC* | 0.951 | C |
| *cyoB* | 0.952 | C |
| *atpG* | 0.954 | C |
| *gltP* | 0.961 | C |
| *sdhC* | 0.963 | C |
| *yihA* | 0.902 | D |
| *rlmE* | 0.923 | D |
| DNA translocase FtsK 4TM domain-containing protein | 0.928 | D |
| *minE* | 0.929 | D |
| *minC* | 0.935 | D |
| *mnmG* | 0.94 | D |
| *ftsW* | 0.944 | D |
| DNA translocase FtsK | 0.944 | D |
| *ftsZ* | 0.945 | D |
| *minD* | 0.946 | D |
| *rodA* | 0.956 | D |
| rod shape-determining protein | 0.96 | D |
| *ftsA* | 0.968 | D |
| *aroE* | 0.892 | E |
| D-alanine--D-alanine ligase family protein | 0.902 | E |
| *ygfZ* | 0.904 | E |
| *dapE* | 0.904 | E |
| *hisIE* | 0.907 | E |
| MFS transporter | 0.912 | E |
| *dapB* | 0.912 | E |
| aminodeoxychorismate-anthranilate synthase component II | 0.912 | E |
| *trpCF* | 0.921 | E |
| *aroB* | 0.922 | E |
| SufS family cysteine desulfurase | 0.923 | E |
| aminodeoxychorismate-anthranilate synthase component II | 0.923 | E |
| *gmhB* | 0.924 | E |
| *trpD* | 0.925 | E |
| *hisD* | 0.925 | E |
| *dapD* | 0.925 | E |
| *leuD* | 0.926 | E |
| *ureC* | 0.927 | E |
| leucyl aminopeptidase | 0.927 | E |
| *glnA* | 0.927 | E |
| DMT family transporter | 0.927 | E |
| *thrC* | 0.929 | E |
| *hisH* | 0.929 | E |
| prephenate dehydratase domain-containing protein | 0.93 | E |
| *pabB* | 0.93 | E |
| urease subunit beta | 0.932 | E |
| *speB* | 0.932 | E |
| *prmC* | 0.932 | E |
| *glyA* | 0.932 | E |
| amino acid aminotransferase | 0.934 | E |
| *metB* | 0.935 | E |
| M3 family metallopeptidase | 0.935 | E |
| *thrB* | 0.936 | E |
| *dapF* | 0.937 | E |
| *cysE* | 0.937 | E |
| *aroC* | 0.937 | E |
| *pabC* | 0.938 | E |
| *metE* | 0.938 | E |
| *metA* | 0.938 | E |
| *asd2* | 0.938 | E |
| *metC* | 0.939 | E |
| *hisC* | 0.939 | E |
| *aroA* | 0.939 | E |
| *aroK* | 0.94 | E |
| *argF* | 0.94 | E |
| *ilvG* | 0.941 | E |
| *aroQ* | 0.941 | E |
| *kdsA* | 0.942 | E |
| *dapA* | 0.942 | E |
| branched-chain amino acid transaminase | 0.942 | E |
| urease subunit gamma | 0.944 | E |
| *ilvD* | 0.944 | E |
| *asd* | 0.944 | E |
| anthranilate synthase component 1 | 0.944 | E |
| *trpA* | 0.945 | E |
| *hisB* | 0.945 | E |
| 3-deoxy-7-phosphoheptulonate synthase | 0.945 | E |
| *lysA* | 0.946 | E |
| *ilvC* | 0.946 | E |
| *ilvA* | 0.946 | E |
| *carA* | 0.946 | E |
| *leuC* | 0.947 | E |
| *cysK* | 0.947 | E |
| *thrA* | 0.949 | E |
| *trpB* | 0.95 | E |
| *leuA* | 0.95 | E |
| phosphoadenylyl-sulfate reductase | 0.951 | E |
| *carB* | 0.952 | E |
| *hisF* | 0.954 | E |
| HisA/HisF-related TIM barrel protein | 0.954 | E |
| *cysD* | 0.956 | E |
| *tyrA* | 0.957 | E |
| *hisG* | 0.957 | E |
| *metF* | 0.964 | E |
| *ilvM* | 0.97 | E |
| UDP-2-3-diacylglucosamine diphosphatase | 0.899 | F |
| *guaA* | 0.919 | F |
| *guaB* | 0.92 | F |
| *dut* | 0.922 | F |
| *udp* | 0.923 | F |
| *ispE* | 0.924 | F |
| *tmk* | 0.926 | F |
| *purH* | 0.928 | F |
| *adk* | 0.928 | F |
| adenylosuccinate synthase | 0.928 | F |
| thymidine kinase | 0.929 | F |
| *purB* | 0.93 | F |
| *orn* | 0.933 | F |
| *gmk* | 0.936 | F |
| *ndk* | 0.939 | F |
| ribose-phosphate pyrophosphokinase | 0.942 | F |
| HIT domain-containing protein | 0.944 | F |
| *pyrH* | 0.946 | F |
| nucleoside transporter C-terminal domain-containing protein | 0.947 | F |
| CTP synthase | 0.948 | F |
| *cmk* | 0.95 | F |
| *nrdA* | 0.952 | F |
| *nrdB* | 0.959 | F |
| *apt* | 0.96 | F |
| *upp* | 0.965 | F |
| MFS transporter | 0.912 | G |
| *tadA* | 0.913 | G |
| *tpiA* | 0.919 | G |
| PTS mannose-fructose-sorbose transporter subunitIIC | 0.925 | G |
| DMT family transporter | 0.927 | G |
| *pgl* | 0.928 | G |
| *pgi* | 0.928 | G |
| inositol monophosphatase family protein | 0.928 | G |
| *gap* | 0.932 | G |
| *gndA* | 0.934 | G |
| *eno* | 0.934 | G |
| phosphoglucomutase | 0.936 | G |
| *pyk* | 0.937 | G |
| *tkt* | 0.94 | G |
| *rpe* | 0.941 | G |
| *manX* | 0.941 | G |
| *pfkA* | 0.942 | G |
| *nagB* | 0.942 | G |
| *hldE* | 0.942 | G |
| *gpmA* | 0.942 | G |
| *nagA* | 0.943 | G |
| *glmM* | 0.943 | G |
| HIT domain-containing protein | 0.944 | G |
| phosphoglycerate kinase | 0.946 | G |
| *ptsI* | 0.949 | G |
| *mdtH* | 0.949 | G |
| *fbaA* | 0.951 | G |
| *zwf* | 0.957 | G |
| HPr family phosphocarrier protein | 0.961 | G |
| 5-formyltetrahydrofolate cyclo-ligase | 0.907 | H |
| *lolB* | 0.909 | H |
| PhoH family protein | 0.913 | H |
| folA | 0.914 | H |
| riboflavin synthase subunit alpha | 0.919 | H |
| *folE* | 0.92 | H |
| *ispA* | 0.923 | H |
| type II toxin-antitoxin system RatA family toxin | 0.924 | H |
| *folP* | 0.924 | H |
| *folC* | 0.927 | H |
| *ubiA* | 0.93 | H |
| *ubiG* | 0.931 | H |
| *pdxH* | 0.931 | H |
| *hemD* | 0.932 | H |
| FAD-dependent monooxygenase | 0.932 | H |
| *coaA* | 0.934 | H |
| *thiI* | 0.935 | H |
| *folK* | 0.937 | H |
| *dxs* | 0.937 | H |
| *ribE* | 0.938 | H |
| *cysG* | 0.938 | H |
| *coaD* | 0.938 | H |
| *pdxA* | 0.939 | H |
| *folB* | 0.939 | H |
| *ribD* | 0.94 | H |
| cell division protein FtsQ-DivIB | 0.94 | H |
| *folD* | 0.942 | H |
| *serC* | 0.943 | H |
| lipoate--protein ligase | 0.943 | H |
| *cyoE* | 0.944 | H |
| UbiX family flavin prenyltransferase | 0.945 | H |
| *ubiB* | 0.945 | H |
| *metK* | 0.945 | H |
| *hemC* | 0.946 | H |
| *coaBC* | 0.946 | H |
| *ribB* | 0.947 | H |
| *ribA* | 0.947 | H |
| IscS subfamily cysteine desulfurase | 0.947 | H |
| *ispB* | 0.949 | H |
| *ubiE* | 0.951 | H |
| *pdxJ* | 0.951 | H |
| *ubiD* | 0.952 | H |
| *ribF* | 0.954 | H |
| *pssA* | 0.921 | I |
| phosphatidate cytidylyltransferase | 0.922 | I |
| 1-acylglycerol-3-phosphate O-acyltransferase | 0.927 | I |
| *fabD* | 0.929 | I |
| *ispC* | 0.93 | I |
| *ybgF* | 0.931 | I |
| SDR family oxidoreductase | 0.933 | I |
| *ispD* | 0.935 | I |
| undecaprenyl-diphosphate phosphatase | 0.936 | I |
| *accC* | 0.937 | I |
| phosphatidylglycerophosphatase A | 0.938 | I |
| *accD* | 0.94 | I |
| *cls* | 0.941 | I |
| *ispF* | 0.943 | I |
| *pgsA* | 0.944 | I |
| *uppS* | 0.946 | I |
| *accB* | 0.947 | I |
| *accA* | 0.947 | I |
| alpha-beta fold hydrolase | 0.948 | I |
| *acpS* | 0.948 | I |
| *ispG* | 0.951 | I |
| *plsX* | 0.955 | I |
| *fabB* | 0.958 | I |
| beta-ketoacyl-ACP synthase III | 0.962 | I |
| *fabZ* | 0.967 | I |
| *acpP* | 0.967 | I |
| *fabA* | 0.977 | I |
| *rpmC* | 0.87 | J |
| Rid family detoxifying hydrolase | 0.874 | J |
| *rplY* | 0.882 | J |
| *rpmG* | 0.893 | J |
| Sua5-YciO-YrdC-YwlC family protein | 0.911 | J |
| *rplO* | 0.914 | J |
| *rpmJ* | 0.915 | J |
| *glyS* | 0.917 | J |
| *rpsT* | 0.918 | J |
| *rplU* | 0.918 | J |
| *rplU* | 0.918 | J |
| *glyQ* | 0.92 | J |
| *rpsI* | 0.921 | J |
| *rplW* | 0.921 | J |
| *rplK* | 0.921 | J |
| *rne* | 0.921 | J |
| *rpsO* | 0.922 | J |
| *rplA* | 0.922 | J |
| *hisS* | 0.922 | J |
| *rplI* | 0.923 | J |
| *rpmE* | 0.924 | J |
| *rplJ* | 0.924 | J |
| *rluC* | 0.924 | J |
| *rplT* | 0.925 | J |
| *glnS* | 0.927 | J |
| *rlmB* | 0.928 | J |
| *fmt* | 0.929 | J |
| *trmD* | 0.931 | J |
| *rlmN* | 0.931 | J |
| *rbfA* | 0.931 | J |
| *metG* | 0.931 | J |
| *efp* | 0.931 | J |
| valine--tRNA ligase 2 | 0.932 | J |
| *tyrS* | 0.932 | J |
| *mepM* | 0.932 | J |
| *infB* | 0.932 | J |
| *infA* | 0.932 | J |
| *argS* | 0.932 | J |
| *thrS* | 0.933 | J |
| *rplF* | 0.933 | J |
| *pheT* | 0.933 | J |
| *murJ* | 0.933 | J |
| *truA* | 0.934 | J |
| *truB* | 0.935 | J |
| *rsmA* | 0.935 | J |
| *rpmA* | 0.935 | J |
| *rplR* | 0.935 | J |
| *map* | 0.935 | J |
| tRNA CCA-pyrophosphorylase | 0.936 | J |
| *rplQ* | 0.938 | J |
| *pnp* | 0.938 | J |
| *rpmD* | 0.939 | J |
| *rpmB* | 0.939 | J |
| *rplM* | 0.939 | J |
| *rplC* | 0.939 | J |
| *leuS* | 0.939 | J |
| *cysS* | 0.94 | J |
| *rplN* | 0.941 | J |
| *ileS* | 0.941 | J |
| *def* | 0.941 | J |
| *alaS* | 0.941 | J |
| *rpsR* | 0.942 | J |
| *gltX* | 0.942 | J |
| *rplP* | 0.943 | J |
| *cysN* | 0.943 | J |
| *aspS* | 0.943 | J |
| *rpsN* | 0.944 | J |
| *rpsM* | 0.944 | J |
| *rplX* | 0.944 | J |
| *rplL* | 0.944 | J |
| *pth* | 0.944 | J |
| *prmB* | 0.944 | J |
| *rplB* | 0.945 | J |
| *rpsF* | 0.946 | J |
| *rplD* | 0.946 | J |
| *rluD* | 0.946 | J |
| *prfA* | 0.946 | J |
| *rpsG* | 0.947 | J |
| *rpsA* | 0.947 | J |
| *rpiA* | 0.947 | J |
| *rimM* | 0.947 | J |
| *rplV* | 0.948 | J |
| *prfB* | 0.949 | J |
| *pheS* | 0.949 | J |
| *serS* | 0.95 | J |
| *rplS* | 0.95 | J |
| *lysS* | 0.95 | J |
| *frr* | 0.95 | J |
| *asnS* | 0.95 | J |
| *rpsS* | 0.951 | J |
| *trpS* | 0.952 | J |
| *tgt* | 0.952 | J |
| *rpsJ* | 0.952 | J |
| *rplE* | 0.952 | J |
| *miaA* | 0.952 | J |
| *rpmF* | 0.953 | J |
| proline--tRNA ligase | 0.953 | J |
| *tuf* | 0.954 | J |
| *rpsK* | 0.954 | J |
| *tsf* | 0.955 | J |
| *rpsC* | 0.955 | J |
| PTS mannose transporter subunit IID | 0.955 | J |
| *rpsL* | 0.957 | J |
| *infC* | 0.957 | J |
| *rpsH* | 0.96 | J |
| *rpsE* | 0.962 | J |
| *rpsD* | 0.962 | J |
| *rpsP* | 0.963 | J |
| *rpsB* | 0.965 | J |
| *rpmI* | 0.965 | J |
| *fusA* | 0.969 | J |
| *rpsU* | 0.972 | J |
| *rpsQ* | 0.977 | J |
| *rpmH* | 0.979 | J |
| biotin--acetyl-CoA-carboxylase ligase | 0.9 | K |
| *rnc* | 0.927 | K |
| transcriptional regulator | 0.93 | K |
| *nusB* | 0.933 | K |
| LysR family transcriptional regulator | 0.933 | K |
| *mntR* | 0.935 | K |
| *slyA* | 0.943 | K |
| *rpoA* | 0.947 | K |
| transglycosylase SLT domain-containing protein | 0.949 | K |
| *nusA* | 0.953 | K |
| *rpoH* | 0.956 | K |
| *rpoB* | 0.956 | K |
| *rpoC* | 0.957 | K |
| *rpoD* | 0.959 | K |
| *greA* | 0.96 | K |
| MarR family transcriptional regulator | 0.961 | K |
| *nusG* | 0.962 | K |
| *rho* | 0.965 | K |
| *rpoZ* | 0.967 | K |
| DNA pol III subunit psi | 0.884 | L |
| *nth* | 0.906 | L |
| *rnpA* | 0.908 | L |
| *dnaX* | 0.914 | L |
| *recD* | 0.918 | L |
| *holA* | 0.918 | L |
| *recC* | 0.919 | L |
| *ssb* | 0.924 | L |
| *rmuC* | 0.924 | L |
| *recB* | 0.927 | L |
| *mutY* | 0.927 | L |
| DNA pol III subunit chi | 0.927 | L |
| *rsmD* | 0.932 | L |
| *rnhA* | 0.932 | L |
| *dnaN* | 0.932 | L |
| *dnaQ* | 0.933 | L |
| DNA pol III subunit delta | 0.934 | L |
| DEAD-DEAH box helicase | 0.934 | L |
| *polA* | 0.939 | L |
| *rnhB* | 0.941 | L |
| *dnaE* | 0.942 | L |
| *xthA* | 0.944 | L |
| *ung* | 0.944 | L |
| OmpH family outer membrane protein | 0.944 | L |
| *ruvX* | 0.946 | L |
| *gyrA* | 0.946 | L |
| *dnaG* | 0.947 | L |
| *gyrB* | 0.949 | L |
| *rnt* | 0.953 | L |
| *dnaB* | 0.953 | L |
| *hns* | 0.954 | L |
| *rppH* | 0.964 | L |
| peptidoglycan DD-metalloendopeptidase family protein | 0.877 | M |
| TonB family protein | 0.895 | M |
| YciC family protein | 0.897 | M |
| *rsmH* | 0.908 | M |
| *lolA* | 0.908 | M |
| FtsX-like permease family protein | 0.908 | M |
| *glmU* | 0.914 | M |
| *rfaD* | 0.915 | M |
| *lptD* | 0.916 | M |
| *murI* | 0.917 | M |
| *ftsL* | 0.917 | M |
| *rseP* | 0.919 | M |
| septum formation initiator family protein | 0.92 | M |
| *lpcA* | 0.92 | M |
| *lgt* | 0.922 | M |
| *waaF* | 0.923 | M |
| *mreC* | 0.926 | M |
| *lnt* | 0.926 | M |
| *ftsI* | 0.926 | M |
| *murB* | 0.928 | M |
| TonB-dependent receptor | 0.929 | M |
| N-acetylmuramoyl-L-alanine amidase | 0.929 | M |
| *kdsB* | 0.93 | M |
| *gutQ* | 0.931 | M |
| *waaC* | 0.933 | M |
| *murE* | 0.933 | M |
| *lptE* | 0.933 | M |
| *ispH* | 0.933 | M |
| *lpxD* | 0.934 | M |
| *lolC* | 0.935 | M |
| *mreD* | 0.936 | M |
| *lolD* | 0.936 | M |
| *ligA* | 0.936 | M |
| *mscM* | 0.937 | M |
| penicillin-binding protein activator LpoB | 0.938 | M |
| *murD* | 0.938 | M |
| *mraY* | 0.938 | M |
| *murF* | 0.941 | M |
| *bamA* | 0.942 | M |
| *murC* | 0.945 | M |
| LPP leucine zipper domain-containing protein | 0.945 | M |
| *murA* | 0.947 | M |
| *mrcB* | 0.948 | M |
| *lpxB* | 0.949 | M |
| *mrdA* | 0.956 | M |
| *lpxA* | 0.956 | M |
| *murG* | 0.959 | M |
| *waaA* | 0.96 | M |
| *lpxC* | 0.963 | M |
| *secE* | 0.91 | N |
| L-D-transpeptidase family protein | 0.912 | N |
| *tolA* | 0.913 | N |
| *yidC* | 0.927 | N |
| *sppA* | 0.932 | N |
| biopolymer transporter ExbD | 0.932 | N |
| *pal* | 0.934 | N |
| *ffh* | 0.935 | N |
| YicC-YloC family endoribonuclease | 0.936 | N |
| *secF* | 0.938 | N |
| *lpxK* | 0.938 | N |
| *tolQ* | 0.941 | N |
| *secA* | 0.941 | N |
| *lspA* | 0.942 | N |
| *secD* | 0.944 | N |
| *lepB* | 0.944 | N |
| *tolB* | 0.945 | N |
| *ftsY* | 0.945 | N |
| *lpxL* | 0.949 | N |
| *leuB* | 0.949 | N |
| *lepA* | 0.95 | N |
| *porin* | 0.956 | N |
| *secY* | 0.959 | N |
| NfuA family Fe-S biogenesis protein | 0.889 | O |
| *trxB* | 0.905 | O |
| *bcp* | 0.909 | O |
| *grxC* | 0.911 | O |
| oxidative damage protection protein | 0.917 | O |
| *dsbB* | 0.917 | O |
| urease accessory protein UreD | 0.918 | O |
| *tsaB* | 0.918 | O |
| SurA N-terminal domain-containing protein | 0.921 | O |
| *grxD* | 0.922 | O |
| UvrD-helicase domain-containing protein | 0.926 | O |
| *hflC* | 0.934 | O |
| *dnaJ* | 0.934 | O |
| urease accessory UreF family protein | 0.936 | O |
| *tsaD* | 0.937 | O |
| *clpX* | 0.939 | O |
| *trxA* | 0.94 | O |
| *ureG* | 0.941 | O |
| *ureG* | 0.941 | O |
| *lon* | 0.942 | O |
| *htpX* | 0.942 | O |
| *grpE* | 0.944 | O |
| *clpB* | 0.944 | O |
| peroxiredoxin C | 0.945 | O |
| *dsbA* | 0.946 | O |
| Do family serine endopeptidase | 0.948 | O |
| *dnaK* | 0.949 | O |
| *hflK* | 0.95 | O |
| *clpP* | 0.95 | O |
| *smpB* | 0.953 | O |
| *groL* | 0.957 | O |
| Hsp20 family protein | 0.959 | O |
| *ftsH* | 0.961 | O |
| co-chaperone GroES | 0.98 | O |
| TusE-DsrC-DsvC family sulfur relay protein | 0.864 | P |
| *cutA* | 0.906 | P |
| MFS transporter | 0.912 | P |
| *zapA* | 0.916 | P |
| *cysC* | 0.925 | P |
| *cysI* | 0.928 | P |
| rhodanese-like domain-containing protein | 0.929 | P |
| *bfr* | 0.93 | P |
| *cysQ* | 0.931 | P |
| Fe-Mn family superoxide dismutase | 0.935 | P |
| sulfate ABC transporter permease subunit | 0.938 | P |
| *cysJ* | 0.938 | P |
| inorganic phosphate transporter | 0.941 | P |
| metal ABC transporter permease | 0.943 | P |
| *cysP* | 0.947 | P |
| *cysA* | 0.947 | P |
| Na-H antiporter | 0.948 | P |
| AI-2E family transporter | 0.95 | P |
| Nramp family divalent metal transporter | 0.954 | P |
| TerC family protein | 0.956 | P |
| *cysT* | 0.957 | P |
| *zur* | 0.958 | P |
| multidrug efflux SMR transporter | 0.958 | P |
| ABC transporter ATP-binding protein | 0.963 | P |
| inverse autotransporter beta domain-containing protein | 0.888 | R |
| MBL fold metallo-hydrolase | 0.903 | R |
| *yigB* | 0.906 | R |
| *coaE* | 0.908 | R |
| LptF-LptG family permease | 0.91 | R |
| LptF-LptG family permease2 | 0.912 | R |
| Trm112 family protein | 0.913 | R |
| *der* | 0.915 | R |
| *tsaE* | 0.916 | R |
| *mnmA* | 0.918 | R |
| SufE family protein | 0.922 | R |
| *sufD* | 0.925 | R |
| *yigL* | 0.926 | R |
| *bamD* | 0.926 | R |
| DMT family transporter | 0.927 | R |
| *tilS* | 0.929 | R |
| *lptF* | 0.929 | R |
| *ybeY* | 0.93 | R |
| *gloB* | 0.93 | R |
| *mnmE* | 0.931 | R |
| *lptB* | 0.931 | R |
| DUF1043 family protein | 0.931 | R |
| *ychF* | 0.933 | R |
| *obgE* | 0.934 | R |
| *mqo* | 0.936 | R |
| *hflX* | 0.938 | R |
| *tldD* | 0.939 | R |
| *pmbA* | 0.94 | R |
| *rsfS* | 0.941 | R |
| HIT domain-containing protein | 0.944 | R |
| *sufB* | 0.946 | R |
| *msbA* | 0.952 | R |
| *nadK* | 0.954 | R |
| YchF-TatD family DNA exonuclease | 0.955 | R |
| *fabG* | 0.955 | R |
| Bax inhibitor-1-YccA family protein | 0.959 | R |
| *rppH* | 0.964 | R |
| *corC* | 0.965 | R |
| *sufC* | 0.967 | R |
| *tmaR* | 0.972 | R |
| 23S | 0.973 | rRNA |
| 16S | 0.985 | rRNA |
| 5S | 0.991 | rRNA |
| *bamE* | 0.891 | S |
| *lptA* | 0.897 | S |
| translocation-assembly module TamB domain-containing protein | 0.917 | S |
| *lptC* | 0.927 | S |
| *dolP* | 0.93 | S |
| *sufA* | 0.934 | S |
| *plsY* | 0.935 | S |
| autotransporter assembly complex family protein | 0.94 | S |
| DedA family protein | 0.942 | S |
| *erpA* | 0.949 | S |
| *yajC* | 0.951 | S |
| YhgN family NAAT transporter | 0.953 | S |
| *hspQ* | 0.956 | S |
| BolA-IbaG family iron-sulfur metabolism protein | 0.882 | T |
| *hfq* | 0.913 | T |
| symmetrical bis-5'-nucleosyl--tetraphosphatase | 0.927 | T |
| *dksA* | 0.947 | T |
| *corA* | 0.954 | T |
| *csrA* | 0.968 | T |
| *cspE* | 0.976 | T |
| *secB* | 0.914 | U |
| *secG* | 0.946 | U |
